# Investigations on the Performance of a 5 mm CdTe Timepix3 Detector for Compton Imaging Applications

**DOI:** 10.3390/s24247974

**Published:** 2024-12-13

**Authors:** Juan S. Useche Parra, Gerardo Roque, Michael K. Schütz, Michael Fiederle, Simon Procz

**Affiliations:** Freiburg Materials Research Center, Albert-Ludwigs-Universität Freiburg, 79104 Freiburg im Breisgau, Germany; gerardo.roque@fmf.uni-freiburg.de (G.R.); michael.schuetz@fmf.uni-freiburg.de (M.K.S.); michael.fiederle@fmf.uni-freiburg.de (M.F.); simon.procz@fmf.uni-freiburg.de (S.P.)

**Keywords:** semiconductors, photon-counting detectors, cadmium telluride, Timepix, Compton camera

## Abstract

Nuclear power plant decommissioning requires the rapid and accurate classification of radioactive waste in narrow spaces and under time constraints. Photon-counting detector technology offers an effective solution for the quick classification and detection of radioactive hotspots in a decommissioning environment. This paper characterizes a 5 mm CdTe Timepix3 detector and evaluates its feasibility as a single-layer Compton camera. The sensor’s electron mobility–lifetime product and resistivity are studied across bias voltages ranging from −100 V to −3000 V, obtaining values of $\mu_{e}\tau_{e}$ = (1.2 ± 0.1) × 10^−3^ cm^2^V^−1^, and two linear regions with resistivities of $\rho_{I}=(5.8\pm 0.2)\;G\Omega \;\mathrm{cm}$ and $\rho_{II}=(4.1\pm 0.1)\;G\Omega \;\mathrm{cm}$. Additionally, two calibration methodologies are assessed to determine the most suitable for Compton applications, achieving an energy resolution of 16.3 keV for the ^137^Cs photopeak. The electron’s drift time in the sensor is estimated to be (122.3 ± 7.4) ns using cosmic muons. Finally, a Compton reconstruction of two simultaneous point-like sources is performed, demonstrating the detector’s capability to accurately locate radiation hotspots with a ∼51 cm resolution.

## 1. Introduction

The 2011 Fukushima Daiichi nuclear accident had significant consequences for political decisions regarding nuclear energy in many countries. For example, Switzerland, Spain, and Japan limited the construction of new nuclear power plants. Similarly, Germany decided to phase out nuclear power, completing its efforts in April 2023 [1]. As a result, a high number of nuclear power plants require proper classification of their nuclear waste. Various classification methods exist that use detectors such as ionization chambers, proportional counters, and gamma spectrometers or cameras for in situ measurements [2]. Given the nature of nuclear decommissioning, space and access restrictions are common, and the lightweight, compact design and high detection efficiency of semiconductor detectors offer advantages in these scenarios [3]. Photon-counting detectors (PCD) are commonly used during the decommissioning process, especially for detecting α particles [4]. They are also used in coded-aperture gamma camera configurations to locate radioactive hotspots [5]. Since coded-aperture gamma cameras suffer from a limited field of view, alternative methods using PCD as part of a Compton camera have been explored [6].

A Compton camera is a detection technique that utilizes Compton kinematics to determine the direction and energy of the γ rays without the need of a collimator. In this technique, the spatial resolution of the detector plays a crucial role; thus, pixelated detectors, such as the Timepix3 [7], serve as a good foundation. The use of a Timepix3 detector in single- and double-layer Compton cameras has been reported [8,9]. A single-layer configuration is advantageous, as it reduces the device size and hardware complexity, offering a field of view of 360°. Typically for this application, the Timepix3 is paired with a cadmium telluride (CdTe) sensor, typically ranging from 1 mm to 3 mm. However, these sensor thicknesses have low interaction efficiency at the photon energies present in a nuclear power plant (0.1 MeV to 1 MeV) as shown in [10]. Therefore, the use of thicker CdTe should be beneficial, as demonstrated by Smolyanskiy et al. [11] for a 5 mm cadmium zinc telluride (CZT) sensor hybridized to a Timepix3.

This work presents the characterization of a 5 mm-thick CdTe sensor (ADVACAM, Prague, Czech Republic) bump-bonded to a Timepix3 detector with a pixel pitch of 55 μm, intended for use as part of a Compton camera device in a nuclear power plant environment. First, measurements describing the behavior of the CdTe crystal are presented, followed by a study of the energy calibration of the full detector assembly. Subsequently, the energy resolution and the timing characteristics of the assembly are examined. Finally, usage of the assembly as a Compton camera to detect radiation hotspots in a controlled environment is demonstrated. The methodologies and results presented are general enough to serve as a basis for other applications using similar equipment.

## 2. Materials and Methods

### 2.1. Timepix3

**Figure 1:**
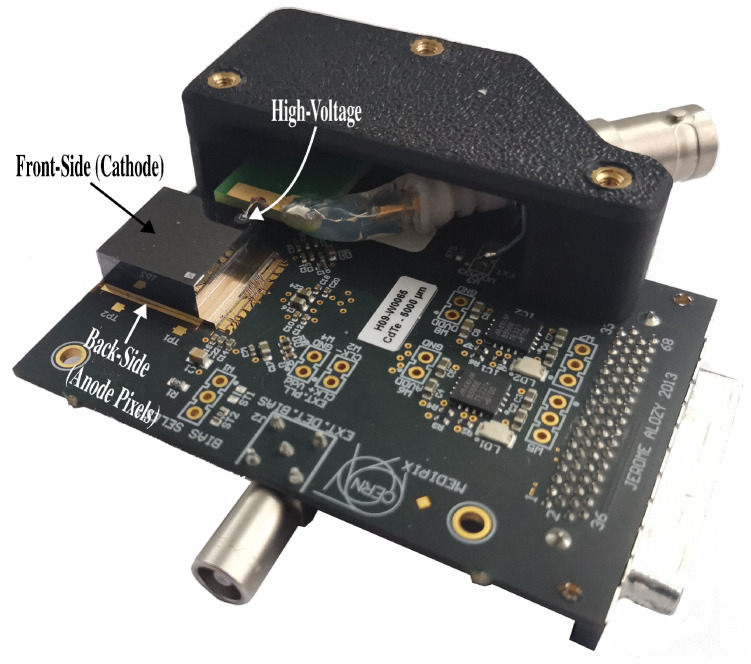
Timepix3 chip with a 5 mm CdTe sensor.

Timepix3 [7] is a hybrid pixel–photon-counting detector (Figure 1) developed by the Medipix collaboration (Geneva, Switzerland). It features a 256 × 256-pixel matrix with a pixel pitch of 55 μm and was developed using 130 nm CMOS technology. The hybrid nature of the detector enables it to be paired with several semiconductor materials, such as Si, CdTe, CZT, or GaAs [12]. In this study, a 5 mm CdTe sensor was bump-bonded with a Timepix3 detector by ADVAFAB [13].

The Timepix3 detector operates in two readout modes. The *frame-based* mode outputs matrix-like data over a user-defined time interval, providing the accumulated number of interactions and the total deposited energy in each pixel. This mode is useful for applications that do not require the measurement of individual particle interactions.

The *data-driven* mode of Timepix3 allows for a constant stream of data output, achieving nearly zero dead time for each pixel. In this mode, the energy and interaction times of single photons are registered. The energy is measured by recording the *time over threshold* (ToT), which refers to the number of clock cycles (40 MHz clock) in which a signal generated by the deposited charge of an incoming particle remains over a user-defined threshold. The proportionality of ToT values to the deposited charge allows them to be converted into the deposited energy of the interaction through calibration, as proposed in [14,15]. The time of arrival (ToA) of the signal is recorded in a 14-bit register at 40 MHz and is refined by a 640 MHz clock, achieving a nominal resolution of 1.5625 ns.

The detector is controlled via USB3 using the AdvaPIX TPX3 readout [16] through the PixetPro 1.8.2 software [17], achieving a maximum data rate close to 20 Mhits s^−1^cm^−2^ with an energy threshold of 5 keV.

### 2.2. Sensor Characterization

General characterization of the CdTe crystal was performed by studying the current–voltage (I–V) behavior, electron mobility–lifetime product ($\mu_{e}\tau_{e}$), and count rate homogeneity of the crystal. It is worth noting that the crystal-detector assembly was actively cooled by a fan in order to maintain a stable temperature.

#### 2.2.1. Current–Voltage Characteristics

The I–V behavior was studied by measuring the variation in the crystal’s leakage current as a function of the bias voltage, in the absence of any photon source, under darkness, and at room temperature (T = 20 °C). The bias voltage was supplied by a high-voltage source (ISEG-THQ) in the range of −100 V to −3000 V, with steps of 50 V. The leakage current was recorded after 30 min at each voltage point in order to ensure thermal stabilization of the detector and a homogeneous electric field in the sensor.

#### 2.2.2. Electron Mobility–Lifetime Product

**Figure 2:**
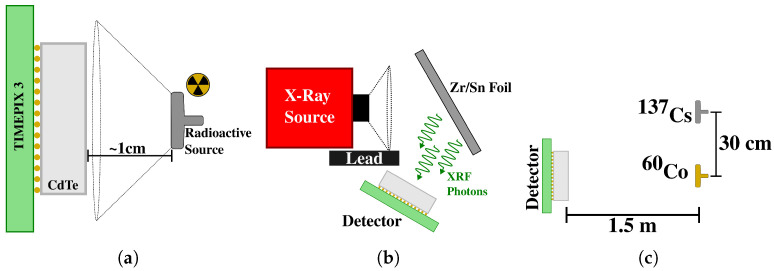
Top view of (**a**) the general setup used to measure a radioactive source spectrum with the Timepix3 detector; (**b**) the XRF measurement setup used for the detector characterization. A lead block was used to shield the detector from the X-rays emitted by the source. (**c**) Setup used for the Compton camera application. Sizes not to scale.

The electron mobility–lifetime product ($\mu_{e}\tau_{e}$) in the crystal was estimated by measuring the ^241^Am photopeak recorded by the detector as a function of bias voltage (−100 V to −3000 V). The radioactive isotope was positioned as close as possible to the sensor in order to maximize photon flux (see Figure 2a). For each bias voltage, a 12 min stabilization period was required to ensure thermal equilibrium in the detector.

Each recorded peak was fitted with a Gaussian function, and the mean values were extracted. These values were then used to fit the Hecht equation, modified to account for the small pixel effect and charge trapping (Equation (1)) [18,19]. This modification assumes that no signal induction occurs while charge carriers are drifting until they reach a region near the contacts (typically with a thickness comparable to the pixel size), where the weighting potential becomes sufficiently high to generate an electrical signal in the detector. Additionally, the electric field in this region is assumed to be linear.

(1) $$E\left(U\right)=E_{0}\;\cdot \;\exp\left(-\frac{(d-D)\;\cdot \;d}{\mu_{e}\tau_{e}\;\cdot \;\left(U-U_{0}\right)}\right)\;\cdot \;\frac{\mu_{e}\tau_{e}\;\cdot \;\left(U-U_{0}\right)}{D\;\cdot \;d}\;\cdot \;\left[1-\exp\left(-\frac{D\;\cdot \;d}{\mu_{e}\tau_{e}\;\cdot \;\left(U-U_{0}\right)}\right)\right]$$

The free parameters from Equation (1) correspond to $\mu_{e}\tau_{e}$, the initial deposited energy of the events ($E_{0}$), and the minimum bias voltage to register a signal ($U_{0}$). The remaining parameters correspond to fixed values given by the experiment such as d=55 μm, corresponding to the pixel size, and D=5mm, the sensor thickness.

#### 2.2.3. Count Rate Homogeneity

The count rate homogeneity of the pixels was measured during 1 h using a ^241^Am source in close proximity to the sensor at a bias voltage of −2000 V. It is worth noting that, unless otherwise mentioned, all other procedures using the detector are performed with this bias voltage.

### 2.3. Energy Calibration

The detector was per-pixel energy calibrated following a protocol similar to those described in [14,15]. The gamma spectrum of the ^241^Am source and the X-ray and gamma spectra of the ^152^Eu source were recorded using a threshold level of 5 keV, with the sources placed in close proximity to the sensor. Each recorded photopeak in each pixel was fitted using a Gaussian function. The mean values of the fitted functions were used to determine the coefficients relating ToT and energy values, and this was achieved employing the following relationship [14]:

(2) $$ToT=a\;\cdot \;Energy+b-\frac{c}{Energy-t}\;,$$

where *a*, *b*, *c*, and *t* are free parameters to be estimated for each pixel.

Given the thickness of the CdTe sensor, photon interactions generate charge-sharing between adjacent pixels in the detector. Thus, the charge of the event is distributed among those pixels, and it is not possible to obtain single-pixel events for low-energy sources, such as ^241^Am. To overcome this problem, it is recommended to perform the calibration from the anode side (back-side) of the detector (see Figure 1 for anode location) [20].

The back-side calibration underestimates low-energy events (≤100 keV) impinging on the sensor from the front-side, as shown in [11]. Thus, the use of front-side (cathode-side) calibration and correcting for charge sharing, employing a clustering algorithm, was explored as an alternative.

Both calibrations were tested by measuring the gamma spectra of ^241^Am, ^152^Eu, ^137^Cs, and ^60^Co, with photons reaching the sensor from the front side (cathode), since it is the most common direction of radiation for Compton camera applications. The deviations of the measured values with respect to the expected values were also calculated.

### 2.4. Detector Characterization

The X-ray fluorescence (XRF) spectra of zirconium (Zr) and tin (Sn) and the spectra of the radiation sources ^137^Cs and ^60^Co were measured to study the energy resolution of the detector in different energy ranges. Each peak was fitted with Gaussian functions, and the energy resolution was estimated using the ratio of the full width at half maximum (FWHM) to the mean energy of the fit. For the XRF measurements, the L10321 microfocus X-ray source [21] from Hammamatsu was employed with a tube voltage of 40 kV and a tube current of 100 μA. Zr and Sn foils were placed in front of the X-ray source, and the detector was placed in such a way that only fluorescence and back scattered photons from the foil could reach it (see Figure 2b).

Additionally, to use this sensor–detector pair in a Compton camera setting, the drift time of electrons in the crystal sensor was required. To estimate this time, measurement of several cosmic muon traces was performed. Each muon penetrated the entire thickness of the sensor from top to bottom; thus, each pixel had a different collection time, depending on the depth [9]. Therefore, the drift time in the sensor corresponded to the time difference between the pixels in the muon track.

### 2.5. Compton Camera Application

To demonstrate the viability of a 5 mm thick CdTe sensor with Timepix3 as a Compton camera, a measurement using two radioactive sources (^137^Cs and ^60^Co) was performed. Both sources were placed 1.5 m from the detector. The ^60^Co source was centered on the detector, and ^137^Cs was displaced 30 cm to the side of the ^60^Co source (see Figure 2c).

The reconstruction of Compton-scattered events inside the detector began by clustering all of the recorded hits. A cluster is a tuple (x,y,E,T), where *x* and *y* are the coordinates in the detector space of the cluster, calculated as the energy-weighted centroid of the hit coordinates. *E* corresponds to the sum of the energies of all the hits in the cluster, and *T* is the ToA of the first clustered hit. Then, only pairs of clusters with a time difference equivalent less than or equal to the drift time of the detector were retained for reconstruction. Therefore, multiple-scatter as well as single-scatter Compton events were discarded.

For each cluster pair, the scattering angle could be calculated using Compton kinematics, which served as a basis for creating a back-projected cone [9]. Using these cones, a back-projection algorithm [22] was implemented to recover the Compton image. The obtained Compton image was deconvolved with a simulated point-spread function (PSF) using the Compton Camera module [23] in GATE [24].

## 3. Results

### 3.1. Sensor Characterization

#### 3.1.1. Current-Voltage Characteristics

**Figure 3:**
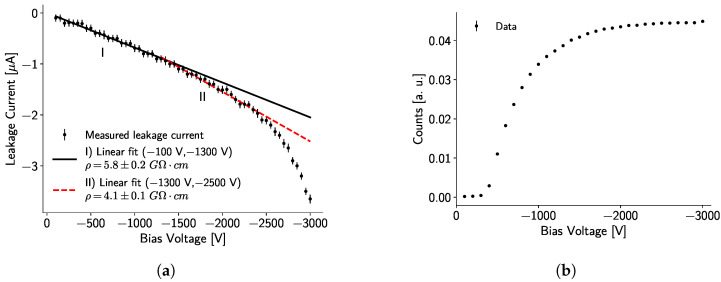
(**a**) Dots correspond to the I–V curve of the CdTe sensor attached to the Timepix3 detector, with error bars accounting for the uncertainty in the measurement. Two linear fits were performed with the data in Zone I (−100 V to −1300 V) and Zone II (−1300 V to −2500 V). These fits are plotted outside the fit range to highlight the difference between the data trends. (**b**) Normalized counts of a ^241^Am radioactive source depending on the bias voltage.

Following the procedure described in Section 2.2, the I–V curve of the 5 mm CdTe sensor was recorded, as shown in Figure 3a. Two linear zones were identified, and a linear fit was performed for each one in the range of −100 V to −1300 V (Zone I) and −1300 V to −2500 V (Zone II). From these linear fits, the resistivity can be estimated as follows:

(3) $$\rho =R\frac{A}{t}\;,$$

where *R* is the electrical resistance, given by the inverse of the slope from the linear fit of the I–V curve; t=0.5cm is the thickness of the sensor; and $A=1.98\;{\mathrm{cm}}^{2}$ is the area of the sensor. The estimated resistivity of the sensor is $\rho_{I}=\left(5.8\pm 0.2\right)\;G\Omega \;\mathrm{cm}$ and $\rho_{II}=\left(4.1\pm 0.1\right)\;G\Omega \;\mathrm{cm}$ for Zones I and II, respectively. Additionally, a superlinear region in which the current increases rapidly appears after −2500 V, indicating the presence of charge carriers injection. The crystal temperature during the experiment was kept at an average value of (28.1 ± 0.1) °C.

In Figure 3b, a visible plateau for the number of registered counts started at −2000 V. This voltage (−2000 V) was then chosen as the operating voltage for the detector because it offered a low leakage current value (∼ −1.5 μA) and enabled maximum recorded counts without causing any stability issues for the detector.

#### 3.1.2. Electron Mobility–Lifetime Product

**Figure 4:**
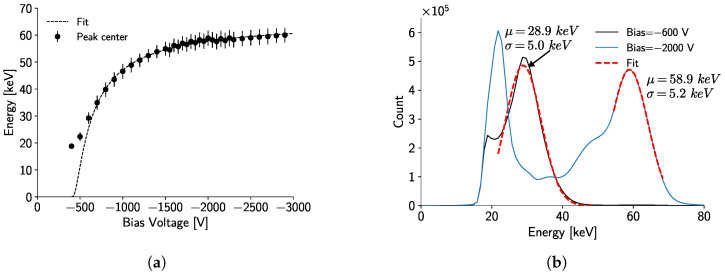
(**a**) Shows the results of the measurements and the fit of the Hecht equation to estimate $\mu_{e}\tau_{e}$. (**b**) Measurement and fit of a ^241^Am peak at a −600 V and −2000 V bias voltage.

The fitting of the Hecht equation is shown in Figure 4a. The data points follow the Hecht function under 1σ; nevertheless, the difference from the fitted model at low bias voltages is given by uncertainties in the estimation of the peak centers due to detector self-fluorescence and by partial charge reconstruction generated by charge sharing. At higher bias voltages, charge sharing is lower, but charge trapping is still noticeable; thus, partial charge collection still occurs.

Figure 4b presents two photopeak measurements for bias voltages of −600 V and −2000 V. It is possible to observe an energy shift in the −600 V peak, which is attributed to the partial collection of electrical charges. Similar peak shifts are observed throughout the full voltage range.

Using the dependence of the fitted mean photopeak values on the bias voltage, the Hecht curve fit estimates the mobility–lifetime product for electrons as $\mu_{e}\tau_{e}$ = (1.2 ± 0.1) × 10^−3^ cm^2^V^−1^. This value is of the same order of magnitude as similar measurements conducted with Timepix and CdTe [25]. Thus, for an operation bias voltage of −2000 V, the expected mean free drift length of charge carriers in the crystal is ∼4.8 cm, which is 9.6 times longer than the thickness of the sensor, implying that the sensor is fully depleted.

Moreover, the minimum voltage required to register a signal is $U_{0}=(-384\pm 21)\;V$, which is consistent with the results shown in Figure 3b, and the fitted initial deposited energy corresponds to $E_{0}=(65.5\pm 0.5)\;keV$. It is worth noting that $E_{0}$ should be expected to correspond to the energy of the ^241^Am photopeak. The observed discrepancy can be attributed to defects in the crystal, which distort the electric field and violate the assumption of a linear electric field. Additionally, charge trapping can significantly reduce the number of collected charges, effectively degrading the detector’s resolution. This effect is not accounted for in Equation (1).

To address these issues, one could propose modeling the electric field inside the crystal based on the known defect locations and incorporating this as a correction to the Hecht equation.

#### 3.1.3. Count Rate Homogeneity

**Figure 5:**
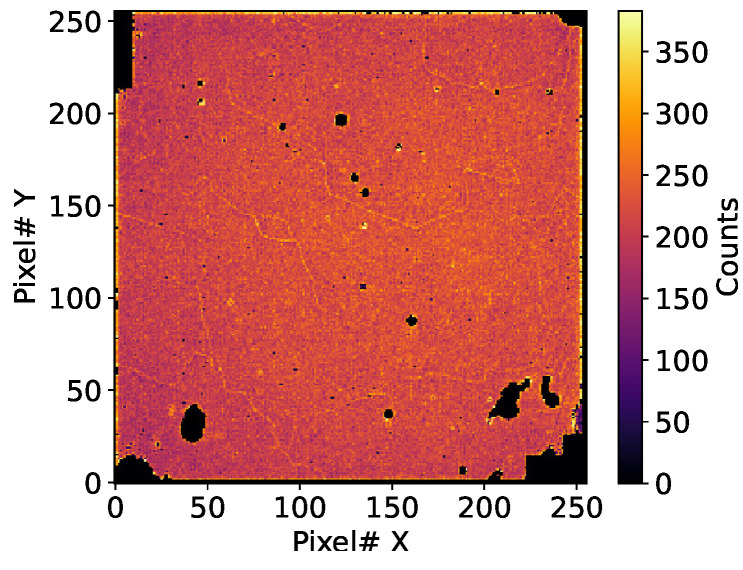
Count rate homogeneity for ^241^Am at $U_{bias}=-2000\;V$.

A flat field image taken with ^241^Am at $U_{bias}=-2000\;V$ is shown in Figure 5. The characteristic vein-like structures of the CdTe crystals are easily identified. In addition, the crystal tended to register more counts around its borders and in the surroundings of crystal defects. Noisy pixels during equalization, calibration, and measurement were discarded and are shown in black. The mean counts per pixel were 217.1 ± 33.2.

### 3.2. Energy Calibration

The detector was per-pixel energy calibrated by measuring the photopeak of a ^241^Am source (59 keV) and the X-ray (∼ 39.9 keV) and gamma (121.2 keV) spectra of ^152^Eu. Since the mean penetration of photons with 59 keV and 121.2 keV in CdTe is 0.2 mm and 1.6 mm, respectively, most photons will interact close to the incident surface of the crystal.

#### 3.2.1. Back-Side Calibration

**Figure 6:**
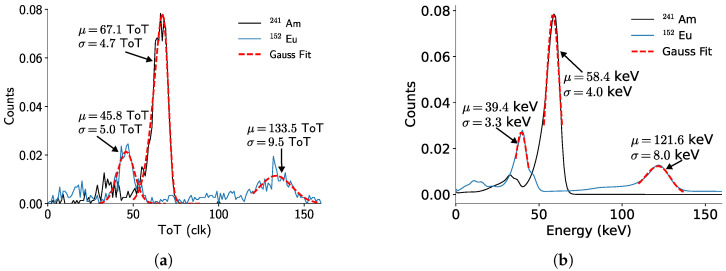
(**a**) Recorded ^241^Am and ^152^Eu raw (uncalibrated) spectra for pixel 37,585. (**b**) ^241^Am and ^152^Eu calibrated spectra for the full detector (anode-side irradiation).

As an example, the fitted uncalibrated spectra for pixel 37,585 are shown in Figure 6a. The fitted values are used to implement the calibration procedure. The calibrated ^241^Am and ^152^Eu spectra are displayed in Figure 6b, obtaining energy resolutions of 4 keV and 8 keV at 58.4 keV and 121.6  keV, respectively.

#### 3.2.2. Front-Side Calibration

**Figure 7:**
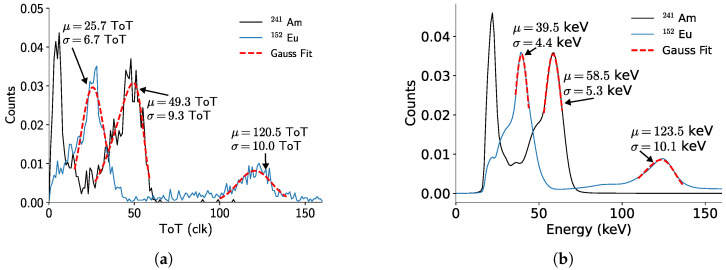
(**a**) Recorded ^241^Am and ^152^Eu raw (uncalibrated) spectra for pixel 37,585. (**b**) ^241^Am and ^152^Eu calibrated spectra for the full detector (cathode-side irradiation).

Given that the irradiation originates from the cathode side, the probability of charge sharing increases significantly. This phenomenon can be seen indirectly from the high contribution of the sensor’s self-fluorescence in the ^241^Am spectrum, at around 23  keV, in Figure 7b. This phenomenon appears in the measured spectrum because the clustering algorithm fails to add the contributions of these fluorescence photons to the main photopeak events.

Similarly to the back-side calibration, an improvement in energy resolution for energies below 200 keV is achieved by the front-side calibration. For example, 5.3  keV at 58.5  keV, which means about 30% less accuracy than the back-side calibration.

#### 3.2.3. Calibrations Comparison

**Figure 8:**
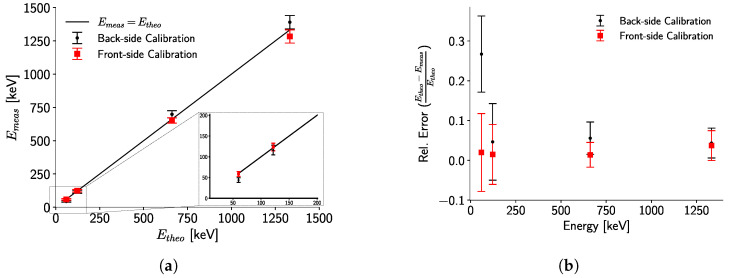
(**a**) Fitted photopeak values for measured ^241^Am, ^152^Eu, ^137^Cs, and ^60^Co radiation incoming from the cathode side of the detector, using both calibrations. The inset shows a zoomed view of the low-energy region, highlighting the better accuracy of the front-side calibration. The continuous line corresponds to $E_{meas}=E_{theo}$. (**b**) Relative error with respect to the expected energy of the photopeaks for each calibration type.

Figure 8 compares the photopeak center values obtained from the spectra of different isotopes using the back-side and front-side calibrations. Each value corresponds to a full detector measurement, where the spectra from all pixels are summed, and the total spectrum is fitted with a Gaussian function. This approach provides a way to study the overall behavior of the detector rather than individual pixels. However, offsets in the per-pixel calibration can degrade the energy resolution, leading to larger error bars.

By comparing the energy resolutions achieved with the two different calibrations, it is possible to see that the back-side calibration is better at correcting the contribution of the sensor self-fluorescence. Nevertheless, as presented in Figure 8a (inset), applying the back-side calibration parameters to photons with energies lower than 200  keV tends to underestimate the photon energy if they reach the detector from the cathode side. This underestimation is mainly due to the incomplete absorption of the photons’ energy in the pixelated contacts. Two factors contributing to charge loss are charge trapping in the sensor layer, whose probability increases with the thickness of the detector, and charge-sharing within neighboring pixels, since pixels with an amount of charge lower than the threshold would not register the event. This effects are more noticeable at the per-pixel level, since the measured photopeaks from the cathode side (Figure 7a) have a higher standard deviation than the ones from the anode side (Figure 6a). However, the full detector spectra have comparable standard deviations.

On the other hand, this underestimation is reduced for higher photon energies (Figure 8a), since they tend to interact closer to the pixelated contacts. The back-side calibration has an average relative error of (10.3 ± 3.7)% (Figure 8b). Removing the lowest energy point reduces the relative error to (4.9 ± 3.7)%.

For the front-side calibration, the average relative error corresponds to (2.0 ± 3.2)%, being more consistent over the complete energy range. The uncertainties are calculated based on the standard deviation from the Gaussian function fitted to each photopeak spectrum.

For the remainder of this work, the chosen energy calibration corresponds to the front-side calibration due to its smaller relative error when measuring photon energies up to 1300  keV. Furthermore, considering that the primary application of this detector is as a Compton camera, the direction of incoming photons will generally be known. Consequently, the detector can be oriented accordingly, allowing the radiation to primarily impact the front side. This ensures better performance with the front-side calibration while still accurately accounting for low-energy photon radiation.

### 3.3. Detector Characterization

#### 3.3.1. Energy Resolution

**Figure 9:**
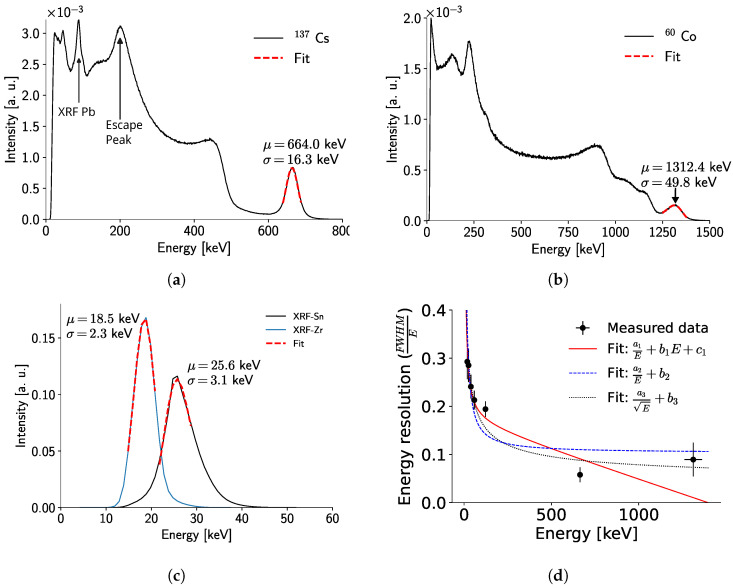
(**a**) ^137^Cs spectrum measured in the detector. (**b**) ^60^Co spectrum. (**c**) Zr and Sn ($K_{\alpha }$, $K_{\beta }$) XRF spectra (**d**) Energy resolution of the detector, calculated from the measurements shown here, and in Figure 7b. Error bars correspond to 1 − σ. The lines show different fitted function to the measured data, with the following values: $a_{1}=2.8\pm 1.3$, $b_{1}=(-1.2\pm 0.5)\times 10^{-4}$, $c_{1}=0.17\pm 0.03$; $a_{2}=4.8\pm 1.4$, $b_{2}=0.10\pm 0.03$; $a_{3}=1.3\pm 0.2$, $b_{3}=0.04\pm 0.03$.

Figure 9a–c show the measured spectra with the full detector and the front-side calibration for ^137^Cs, ^60^Co, and the XRF of Zr and Sn, which correspond to overlaps of their respective $K_{\alpha }$ and $K_{\beta }$ fluorescence lines.

For the radioactive isotopes, the deviation of the fitted mean value from the expected photopeak energy is less than 1%. It is worth noting that the measurement of the ^60^Co photopeak at 1332  keV is affected by the proximity of the photopeak at 1173  keV, which broadens the peak by adding counts in the region between the two photopeaks, thus deteriorating the detector’s estimated energy resolution at this energy (Figure 9d). Nonetheless, the peak is resolved, implying an improvement over previous work with a detector of similar thickness [20]. In both spectra (Figure 9a,b, a peak at around 90  keV, corresponding to the XRF of lead (Pb) used as shielding, is observed.

Figure 9d shows the behavior of the detector’s energy resolution up to 1312  keV. The resolution improves with energy, a trend already observed elsewhere [26,27,28], except for the highest energy point. This anomaly is a result of the presence of the second photopeak for ^60^Co, which is not resolved under the Compton continuum and edge.

Three different functions were fitted to the detector resolution data in Figure 9d. The first model ($\frac{a}{E}+bE+c$), based on previous work [26], provides good accuracy below 200  keV but fails at higher energies due to linear energy dependence. The second model ($\frac{a}{E}+b$) eliminates linear dependency but sacrifices low-energy accuracy, describing resolution as nearly constant above 200  keV, which does not match the data trend. The third model ($\frac{a}{\sqrt{E}}+b$) shows the most promise. It describes the low-energy range reasonably well and aligns with high-energy data points within three standard deviations, indicating statistical consistency with the observed trend. Future work should address the lack of data points in the 200 keV to 1500 keV range in order to develop a comprehensive detector resolution model.

The X-ray fluorescence of Zr shows a deviation from its expected value (∼15.7 keV) of 18%. This deviation is explained by several factors, the principal one being that this energy value is close to the detector’s threshold (∼5 keV), and the calibration used is insufficient to describe the detector’s behavior around this point, since no calibration point near the threshold was measured to correctly fit the surrogate function in Equation (2). Additionally, this value is outside of the calibration range, and the extrapolation is unreliable since a minimal variation in ToT implies a considerable variation in energy. In contrast, the deviation of the XRF of Sn is ∼1% (Figure 9c). Thus, for this detector, energy measurements higher than 25  keV are reliable if they impinge on the sensor from the cathode side.

#### 3.3.2. Drift Time

**Figure 10:**
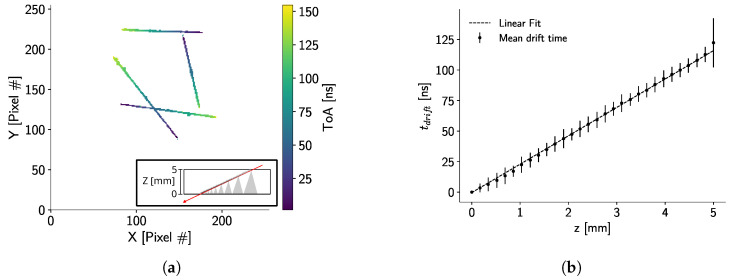
(**a**) Muon tracks measured in the detector. Inset depicts the expected direction of both a muon track traversing the full sensor thickness and the electron clouds generating by each pixel deposition. (**b**) Mean values of the *z* coordinates vs drift times. The dotted line represents a linear fit, and the error bars correspond to 1 − σ.

Characterizing the drift time of electrons through the full sensor thickness is vital for any Compton camera application with this detector assembly. Thus, measurement of atmospheric muons was performed for an experimental estimation. Over these tracks, geometrical constraints were imposed to ensure the track goes through the full sensor thickness. Valid tracks should have at least 150 contiguous pixels and should be 30 pixels away from the sensor boundaries. A selection of muon tracks used for the calculation is shown in Figure 10a.

Based on the geometrical approach shown in [29], the calculation of the *z* coordinate of each point on the track is based on an interpolation between its *x* and *y* coordinates. Let $P_{in}=\left(x_{in},y_{in}\right)$ be the point on the track with the maximum ToA value and $P_{out}=\left(x_{out},y_{out}\right)$ be the point with the minimum ToA value. Now, the *z* coordinate of a point P=(x,y) in the track is given by

(4) $$z\left(P\right)=\frac{d}{|P_{out}-P_{in}|}\times \left|P-P_{in}\right|\;,$$

where *d* is the sensor thickness (*d* = 5 mm). Then, each *z* coordinate can be related to the ToA values of point *P*. For reference, *z* = 5 mm corresponds to the sensor cathode.

Figure 10b shows the mean values of the *z* coordinates and drift times calculated for all 470 muon tracks. For this plot, the binning of the *z* coordinates is 0.167 mm. Based on this calculation, on average, the drift time of an electron through the full sensor thickness is equal to (122.3 ± 7.4) ns. Assuming a homogeneous linear electric field, the theoretical drift time of electrons is given by

(5) $$\tau_{drift}=\frac{-d^{2}}{\mu_{e}\times U_{bias}}\;.$$

For a bias voltage of −2000 V, the drift time is equal to 119 ns, which is approximately 3% lower than the experimental value. This discrepancy is expected due to defects in the crystal. For example, the experimental drift time for a 2 mm-thick CdTe sensor in [9] is reported to be 86 ns, deviating 2% from the theoretical value of 84 ns.

The data point at 0.5 cm (Figure 10b) does not follow the linear trend due to the selection criteria for the entry point ($P_{in}$). Usually, around this point, the energy deposited by the muon is shared between several pixels, and the ToA and *x*, *y* coordinates for point $P_{in}$ have to be calculated as the energy-weighted average of the surrounding pixels. This introduces a bias towards high-energy depositions, which do not directly relate to the point of entry of the muon in the detector. Nevertheless, this point is included as part of the analysis, since virtually every muon track shows this behavior.

### 3.4. Compton Camera Application

**Figure 11:**
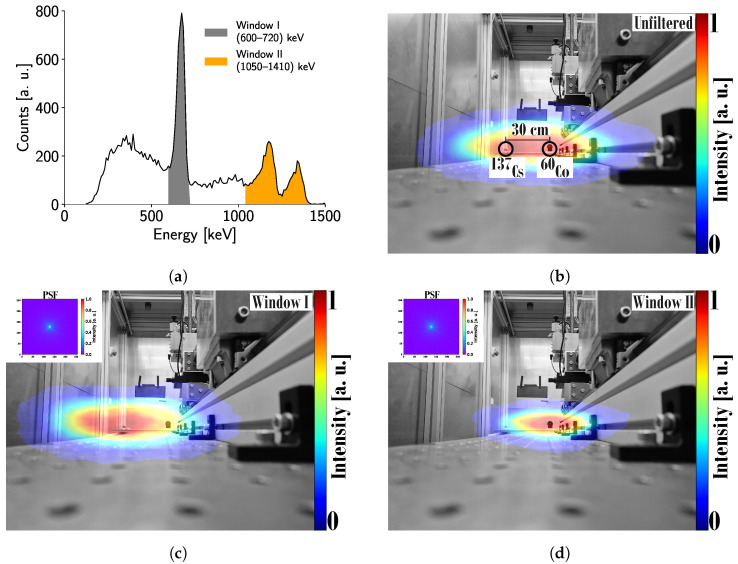
(**a**) Energy spectrum of the reconstructed Compton events. The highlighted energy windows are used to filter the events in (**c**,**d**). Window I corresponds to the range 600 keV to 720 keV, while Window II corresponds to 1050 keV to 1410 keV. (**b**) Compton image reconstructed from all the events, without any filtering. (**c**) Compton image reconstructed using only events in Window I and deconvolved using the PSF shown in the inset. (**d**) Reconstructed Compton image for events with energy in Window II. The inset shows the PSF used for deconvolution.

Figure 11b presents the Compton image reconstructed from the experimental setup described in Section 2.5, where only a single radiation spot is visible. The FWHM of the radiation spot is approximately 87 cm 1.5 m from the detector. This result is dependent upon the geometrical construction of the experimental setup, and thus, will change if the sources are located at a different distance.

It is worth noting that, for this study, the activities of the sources are very similar: 3.6 MBq and 3.3 MBq, for ^60^Co and ^137^Cs, respectively. Consequently, they contribute almost equally to the Compton image. However, if the activities differ significantly, this could worsen the localization resolution. The principal factors affecting the FWHM in this experiment include partially reconstructed events contributing to the Compton continuum in the spectrum (Figure 11a); the detector’s energy resolution, which limits the accuracy of the scattering angle estimation; and the reconstruction algorithm used. For future industrial applications, more accurate reconstruction algorithms should be explored. Additionally, the energy resolution and measurement accuracy could be improved by maintaining a constant detector temperature [30].

As the photopeaks of the different sources are clearly identified in Figure 11a, the resolution of the individual sources at 1.5 m can be analyzed. Figure 11c shows an FWHM of approximately 76 cm for the ^137^Cs source, while Figure 11d shows an FWHM of approximately 51 cm for the ^60^Co source. These FWHM values explain why only one radiation spot is visible in Figure 11b, as the spots overlap when the separation between the sources is only 30 cm. Therefore, separating the sources by a distance greater than approximately 76 cm should result in two distinct radiation spots under the same experimental conditions.

It is also noteworthy that, following the back-projection reconstruction, the images were deconvolved using a simulated point-spread function (PSF), shown as insets in Figure 11c,d, of a 5 mm CdTe crystal with a 100% efficiency detector. These deconvolved images demonstrate enhanced localization resolution.

## 4. Discussion and Conclusions

The CdTe crystal exhibits detector-grade (>0.1 GΩ cm) resistivity for a bias voltage between −100 V to −2500 V, and two linear zones were identified (−100 V to −1300 V and −1300 V to −2500 V). Additionally, a superlinear region appears for voltages higher than -2500 V. The low-bias voltage zone, with a resistivity of $\rho_{I}=\left(5.8\pm 0.2\right)G\Omega \;\mathrm{cm}$, is not recommended as an operating voltage for this thick crystal, since incomplete collection of charge carriers occurs, as shown in Figure 3b. Thus, for this detector, it is recommended to operate in the range of −1300 V to −2500 V, which shows a linear variation of the leakage current with respect to the bias voltage, with a resistivity of $\rho_{II}=\left(4.1\pm 0.1\right)G\Omega \;\mathrm{cm}$. This resistivity is on the same order of magnitude as those reported for other CdTe crystals [31,32].

For a thick crystal like the one used in this work, over-depleting the sensor is recommended. However, due to some edge defects (visible in Figure 5), the maximum stable operation voltage of the detector was found to be around −2000 V, offering complete charge collection and being sufficiently far from the superlinear region. It is worth noting that during acquisition and in the presence of radiation, the leakage current for a given bias voltage tends to increase. Thus, the detector can reach the superlinear region under these conditions, becoming unstable.

The reported mobility–lifetime product for electrons ((1.2 ± 0.1) × 10^−3^ cm^2^V^−1^) in CdTe is consistent with values reported in the literature (∼1 × 10^−3^ cm^2^V^−1^ [25,31]). At bias voltages below 700 V, the fit of the Hecht function is affected by charge-sharing between neighboring pixels and crystal self-fluorescence, leading to an incorrect reconstruction of the photopeak. At higher bias voltages, the presence of crystal defects generates charge trapping and modifies the electric field, affecting the expected plateau of the Hecht equation. This effect can be observed in Figure 4a at around 2000 V. While different models for the electric field inside the Hecht equation could potentially correct these defects, such an investigation is beyond the scope of this work. Additionally, future work should investigate the same properties on a different 5 mm-thick CdTe sensor to determine whether the observed behaviors are intrinsic to the crystal or the Timepix3 chip.

Concerning the detector energy calibration and response, it was found that when using the device as part of a Compton camera, where the direction of the incoming radiation is known to some degree, it is beneficial to use a front-side per-pixel calibration. This approach achieves an average error in the estimation of energy of (2.0 ± 3.2)%, compared to (10.3 ± 3.7)% using the alternative back-side per-pixel calibration, and also avoids overestimating the photon’s energy for values <200 keV. Nevertheless, as presented in Figure 9c, the front-side calibration shows a considerable deviation from the expected energy values for energies <25 keV. This behavior is due to the lack of calibration points around the threshold (5 keV) of the detector. Also, due to the thickness of the sensor, charge-sharing increases significantly for low photon energies, leading to events being partially reconstructed by the clustering algorithm. Thus, in order to use this crystal thickness for measuring low-energy photons, a calibration focusing on the specific low-energy region should be performed.

The possibility of using a Timepix3 detector with a 5 mm-thick CdTe sensor is demonstrated in Figure 11b. A localization resolution of approximately 87 cm is achieved for two point-like radiation sources located 1.5 m from the detector and separated by 30 cm. Under these experimental conditions, the localization resolution for each radiation source corresponds to ∼76 cm and ∼51 cm for ^137^Cs and ^60^Co, respectively. Thus, only one radiation spot is visible in Figure 11b, since, without any energy filtering, both FWHM will overlap.

The obtained resolutions align with reported literature values. In [33], a 20° FWHM value is obtained for a single-layer Compton camera using CdTe, which, for this work’s experimental geometry, is equivalent to ∼55 cm. However, these systems are far from current state-of-the-art gamma cameras using coded aperture masks, which have a resolution around 5° [34]. The primary limitation on localization resolution is the accuracy of Compton angle reconstruction, which depends on energy measurement precision and event selection. Energy resolution can be improved by modeling detector response through Monte Carlo simulation and deconvolving this response from experimental measurements. To address event selection accuracy, an empirical method based on event scattering probability, such as that proposed in [35], could be implemented. Alternatively, machine learning methods for modeling the detector, such as deep learning, could be explored.

For this proof-of-concept, a simple back-projection algorithm was used. Future work should explore alternative methods offering better localization resolution, such as filtered back-projection [36,37] or maximum likelihood–expectation maximization [38].

Improving localization resolution while maintaining event reconstruction speed and system compactness is crucial for future research and industrial applications, such as nuclear power plant monitoring or emergency response to natural disasters. In these scenarios, size constraints often take precedence over precise radioactive source localization. The combination of a thick CdTe crystal with a Timepix3 chip, along with the techniques demonstrated in this work, could be a valuable solution for use as the detection system in a compact Compton camera.

## Data Availability

The raw data supporting the conclusions of this article will be made available by the authors on request.
